# DNA Methylation Patterns in the Social Spider, *Stegodyphus dumicola*

**DOI:** 10.3390/genes10020137

**Published:** 2019-02-12

**Authors:** Shenglin Liu, Anne Aagaard, Jesper Bechsgaard, Trine Bilde

**Affiliations:** Department of Bioscience, Aarhus University, 8000 Aarhus C, Denmark; Liu.Shenglin@bios.au.dk (S.L.); anneaagaard@bios.au.dk (A.A.); Jesper.Bechsgaard@bios.au.dk (J.B.)

**Keywords:** DNA methylation, gene expression, epigenetics

## Abstract

Variation in DNA methylation patterns among genes, individuals, and populations appears to be highly variable among taxa, but our understanding of the functional significance of this variation is still incomplete. We here present the first whole genome bisulfite sequencing of a chelicerate species, the social spider *Stegodyphus dumicola*. We show that DNA methylation occurs mainly in CpG context and is concentrated in genes. This is a pattern also documented in other invertebrates. We present RNA sequence data to investigate the role of DNA methylation in gene regulation and show that, within individuals, methylated genes are more expressed than genes that are not methylated and that methylated genes are more stably expressed across individuals than unmethylated genes. Although no causal association is shown, this lends support for the implication of DNA CpG methylation in regulating gene expression in invertebrates. Differential DNA methylation between populations showed a small but significant correlation with differential gene expression. This is consistent with a possible role of DNA methylation in local adaptation. Based on indirect inference of the presence and pattern of DNA methylation in chelicerate species whose genomes have been sequenced, we performed a comparative phylogenetic analysis. We found strong evidence for exon DNA methylation in the horseshoe crab *Limulus polyphemus* and in all spider and scorpion species, while most Parasitiformes and Acariformes species seem to have lost DNA methylation.

## 1. Introduction

DNA methylation, a form of epigenetic modification of the genome, is a widespread phenomenon across the animal kingdom, but it is evident that methylation patterns and their function and molecular mechanisms vary [1]. Some of the proposed functions of DNA methylation are to regulate the level of gene expression, differential splicing, and DNA structure [1,2]; therefore, DNA methylation supposedly plays an important role in development, differentiation, and potentially in adaptation [3,4]. This implies that DNA methylation has the potential to add an additional layer of information to the DNA sequence, a layer that can potentially be stored within and across generations [5].

The patterns, functions, and mechanisms of DNA methylation are divergent among taxonomical groups. For example, vertebrates appear to be heavily methylated across their entire genomes (globally), and DNA methylation functions to downregulate gene expression, aid DNA structure, guide differential splicing, and silence transposable elements (TEs) [2]. Invertebrate genomes, on the other hand, are primarily methylated in gene bodies, and the main function is thought to involve upregulation of gene expression [1,6,7], and a function of stabilization of gene expression has recently been proposed [8]. In arthropods, patterns of DNA methylation have mostly been studied in pancrustacean species, and have been found to be highly diverse. Within insects, there is strong phylogenetic divergence of the occurrence of DNA methylation among orders. For example, all studied Odonata and Thysanoptera species have DNA methylation, all studied Diptera species lack DNA methylation, and variable occurrence of DNA methylation is found in Hymenoptera and Coleoptera species [9]. The occurrence of DNA methylation is tightly linked to the presence of the molecular machinery catalyzing DNA methylation. A family of enzymes, DNA methyltransferases (DNMTs), catalyze the addition of a methyl group to carbon-5 of cytosine residues. Two main groups of DNMTs exist; DNMT3s catalyze de novo methylations, while DNMT1s maintain methylation patterns during cell divisions [10]. A third member of the family DNMT2 that was initially considered a DNMT has since been shown to be a tRNA methyltransferase [11]. The majority of methylated cytosines in animal genomes are found in CpG context (>99%) [12,13]. With only few exceptions, all insect genomes that have DNA methylation also carry one or more copies of DNMT1, while many do not carry DNMT3 [9].

Gene methylation across invertebrate genomes varies; some genes are not methylated at all, while others vary in the degree of methylation. Genes that are highly methylated are predominantly housekeeping genes [6,14], which are defined by being constitutively expressed. Housekeeping genes are often under stronger evolutionary constraints than other genes [15,16], due to a limited number of possible neutral and/or beneficial mutations that can take place. Accordingly, more mutations will be deleterious in housekeeping genes and therefore be removed by purifying selection. The non-synonymous evolutionary rates of protein coding genes are consequently predicted to be lower in housekeeping genes, and since housekeeping genes are generally more methylated, evolutionary rates of housekeeping genes can be predicted to correlate to the extent of DNA methylation [17]. In chelicerate species, the current knowledge of patterns and functions of DNA methylation is sparse. In the spider mite, *Tetranychus urticae*, there is experimental validation of a low level of DNA methylation from a low number of protein coding genes, and indirect evidence of genome-wide DNA methylation [18]. It is, however, unknown if DNA methylations is restricted to protein coding genes or widespread across chelicerate genomes, and which function they serve, if any.

Here we investigate genome-wide DNA methylation distribution patterns in the spider species *Stegodyphus dumicola*, using whole genome sequencing (WGS) and whole genome bisulfite sequencing (WGBS). In combination with RNA sequence data, we investigate the role of DNA methylation in gene regulation. Methylated cytosines are prone to spontaneous deamination (C -> T) [19,20], thereby causing genomic regions that are methylated or have a recent history of being methylated to have fewer CpGs than expected based on cytosine and guanine frequencies. The measure CpG _O/E_ (observed/expected), as a proxy for the extent of methylation, is frequently applied [1,9], and allows us to indirectly infer patterns of DNA methylation across genome sequences without directly determining DNA methylation. Taking advantage of the CpG _O/E_ measure and the genome sequence of the closely related species *Stegodyphus mimosarum*, we examine how conserved DNA methylation patterns are between these two social *Stegodyphus* species, and correlate DNA methylation level to evolutionary rates of protein coding genes. In addition, we investigate the presence and pattern of DNA methylation in other chelicerate species whose genomes have been sequenced, in a comparative phylogenetic context by searching their genomes for genes encoding DNMTs and estimating CpG _O/E_ as a proxy for current or recent historical DNA methylation.

## 2. Materials and Methods

### 2.1. Study Species

The spider genus *Stegodyphus* contains more than 20 species, and three of them have independently evolved social behavior [21]. The three species share some common characteristics such as inbreeding, a female biased sex ratio, and strong extinction/recolonization dynamics [22,23]. These traits cause an extremely low species-wide genetic diversity within species [24,25]. Particularly, *S. dumicola* has one of the lowest genetic diversities estimated in any species studied so far [25,26].

### 2.2. Sample Collections and Datasets

Four datasets were created in this study. These are PacBio whole genome sequencing data (hereafter referred to as WG-PB), Illumina whole genome sequencing data (WG-I), transcriptome sequencing data (RNA-seq), and whole genome bisulfite sequencing data (WGB). WG-PB and WG-I were used to assemble a reference genome for *S. dumicola*. RNA-seq was used to aid the prediction of protein-coding genes in the reference. WGB was used to investigate the context and the distribution of cytosine methylation in the *S. dumicola* genome. It was also used in combination with the RNA-seq data to examine the functional role of methylation in gene expression.

All the datasets have their sample sources from six populations of *S. dumicola* in Southern Africa (Figure S1, Table S1). For WG-PB, we collected 50 individuals from a single nest from the Etosha population. For WG-I, 90 individuals were collected from all six populations, specifically 15 nests (family groups) per population and one individual per nest. The RNA-seq data came from an experimental design involving four populations (Etosha, Stampriet, Betta, and Karasburg), each with 10 nests. Fifty individuals from a single nest of a population were split into five groups, each being acclimated to a different raising temperature in the lab (15 °C, 19 °C, 23 °C, 25 °C, and 29 °C, respectively). Different acclimation temperatures were used to maximize the total number of transcripts expressed in order to obtain the best protein coding gene annotation possible (see below). For each acclimation, we set 10 replicates using the 10 nests. This eventually amounted to a total of 200 experiments (4 populations × 5 temperatures × 10 replicates). One individual from each experiment was chosen for transcriptome sequencing. The WGB data came from the same experiment set as RNA-seq. Here we chose individuals (one per experiment) from 20 experiments involving two populations (Betta and Karasburg) and one temperature (25 °C). The Betta and Karasburg populations differ by several climatic parameters, and especially in humidity and temperature, with Karasburg being dryer and colder than Betta (www.worldclim.org).

### 2.3. Whole Genome Sequencing, Assembly, and Annotation

#### 2.3.1. DNA Extraction and Sequencing

To generate the WG-PB data, we first extracted genomic DNA from the pool of 50 individuals from a single nest. We note that intra-colony genetic diversity is extremely low in *S. dumicola* [25], so nucleotide diversity, copy number, and structural variation should not influence the genome assembly. The spiders were flash frozen in liquid nitrogen and ground to a powder before adding 10 mL of extraction buffer (10 mM Tris pH 8, 100 mM EDTA, 0.02 mg RNase/mL buffer, 0.5% SDS). After incubation at 37 °C for 1 h, 50 μL of proteinase K (20 mg/mL) was added, and the sample was incubated in a 50 °C water bath for 3 h. The sample was equilibrated to room temperature before 10 mL of phenol was added. After mixing gently for 10 min, the sample was centrifuged for 15 min at 3000 rpm. The viscous aqueous phase was transferred to a new tube using a wide-pore glass pipette. Phenol extraction was repeated twice. Two milliliters of ammonium acetate (10 M) was added, and the sample was mixed gently. After adding 2 volumes of ethanol at room temperature, DNA was collected using a bended pipette tip, air-dried for about 10 min, and dissolved in a TE buffer. The DNA was sequenced on six SMRT cells, resulting in 37.2 Gb of data. The N50 of the sequencing reads was 15.5 Kb. We filtered out reads shorter than 1000 bp, and 99.4% of the data remained. PacBio data was produced by the Duke Center for Genomic and Computational Biology (NC, USA).

For the WG-I data, we extracted genomic DNA from 90 individuals separately; fifteen individuals from separate nests from five Namibian populations and one South African population, using the DNeasy Blood and Tissue kit from Qiagen (Hilden, Germany). The 15 DNA samples from each population were pooled in equal concentrations before library construction (300 bp insert size) and sequenced on a HiSeq2500 platform. In total, 262 Gb of paired-end sequencing data were generated from the six libraries with a read length of 150 bp. The data was filtered before genome assembly. Each read was trimmed off by the first 10 bp and the last 20 bp. Reads containing more than five Ns or containing polyAs longer than 27 bp were discarded. Reads containing more than 10 nucleotides with a phred score lower than 20 were also discarded. After filtering, 178 Gb of data remained (67.9%). Illumina data was produced by Novogene (Hongkong).

#### 2.3.2. Genome Assembly

We adopted a hybrid assembly pipeline DBG2OLC to assemble the genome [27], which allows for a combination of long read and short read data. First, a set of short but accurate contigs (16,106,583 contigs with an N50 of 1053 bp and a total length of 2,916,046,763 bp) was constructed from the WG-I data using a DBG-based (De Bruijn graph) assembler, SparseAssembler [28]. The contigs were filtered according to depth (>14 and <40) and length (>300 bp) (Figure S2) (retaining 1,114,826 contigs with an N50 of 2427 bp and a total length of 1,764,852,519 bp). Our experimental runs revealed that filtered contigs helped increasing the N50 and the total length of the final assembly (Table S2). The filtered contigs and the WG-PB data were input into DBG2OLC to generate a draft assembly. The key parameters of the program were set as k 17, AdaptiveTh 0.001, KmerCovTh 2, MinOverlap 20, RemoveChimera 1. The value of each parameter was fine-tuned through experimental runs, aiming for a draft assembly of a high N50 and a large length (Table S2). The draft assembly was polished with the WG-I data using Pilon [29].

Two methods were used to assess the quality of the assembly. First, we ran an ortholog search using BUSCO v3.0.2 [30] against the Arthropoda_odb9 database. This database records 1066 orthologs found among arthropods. A high recovery rate of the orthologs could indicate the completeness of the assembly. Second, we mapped the raw Illumina reads of WG-I to the assembly using BWA v0.7.15 [31] and inspected the mapping rate and the normality of the depth distribution and the insert size distribution. 

#### 2.3.3. Genome Annotation

Genes were predicted using AUGUSTUS v3.2.2 [32]. First, the orthologs recovered from the BUSCO analysis were used to retrain AUGUSTUS for a set of gene-predicting parameters that are specific to the *S. dumicola* genome. Untranslated regions (UTR) predictions were allowed. Next we used the obtained parameters to predict the genes in the assembly. Splice sites identified from the RNA-seq data (see below) (depth >50) were incorporated into the process as hints to aid the prediction. The quality of the prediction was evaluated by comparing the exons discovered by the RNA-seq data and those predicted by AUGUSTUS. The predicted genes were annotated both by using InterProScan5 and by blasting against UniRef90 database.

We used RepeatModeler and RepeatMasker (version 3.3.0) [33] to identify and mask repeat content of the genome assembly. We initially built a repeat library using Tandem Repeat Finder (TRF) (version 4.04) [34], RECON (version 1.07) [35] and RepeatScout (version 1.0.5) [36], which are implemented in RepeatModeler (version 1.0.5). We subsequently used RepeatMasker to screen and softmask the genome assembly for the identified tandem repeats, interspersed repeats, and low complexity sequences.

### 2.4. Gene Expression

#### RNA Extraction and Sequencing

One individual from each lab acclimated nests was used for individual RNA expression analyses, resulting in 10 replicates per population/acclimation group. RNA was extracted using QIAGEN RNeasy Mini Kit (Qiagen, Hilden, Germany), following the manufacturer’s instructions, adding the amount of extraction buffer corresponding to spider size. RNA was successfully extracted and sequenced from 199 of the 200 spiders. Libraries were constructed on each RNA sample separately using NEBNext Ultra TM RNA Library Prep Kit (New England Biolabs, Ipswich, MA, USA) for Illumina, and 150 bp paired end sequencing was performed on an Illumina HiSeq2500 platform. Library construction and sequencing were performed by Novogene (Hongkong).

### 2.5. Whole Genome Bisulfite Sequencing

#### 2.5.1. DNA Extraction and Sequencing

DNA was extracted from one individual from each nest originating from Betta and Karasburg that were acclimated to 25 degrees using the DNeasy Blood and Tissue kit from Qiagen (Hilden, Germany), and pooled in equal concentration from each population before bisulfite treated and Illumina sequenced on a HiSeq2500 platform (150 bp paired-end). λDNA was used as a control for a bisulfite conversion rate, and 99% of the unmethylated cytosines were converted. In total, 200 Gb of data were obtained.

#### 2.5.2. Mapping and Methylation Calling

We used Bismark v0.19.0 [37] to map the bisulfite sequencing reads to the *S. dumicola* reference genome, and to call the methylated sites. First, the reads were quality-checked using FastQC v0.11.5 [38] and were filtered using Trim Galore v0.4.1 [39] by allowing “--trim1.” The reference genome was indexed using “bismark_genome_preparation” in the Bismark package by invoking bowtie. The mapping was conducted using default parameters. We inspected the depth distribution, insert size distribution, and mapping rate (Figure S3, Table S3). We subsequently ran “bismark_methylation_extractor” and “bismark2bedGraph” to extract all the C cites covered by the sequencing reads together with their methylation status. The first two base pairs of all the Read 2 files were removed based on the M-bias plots. We included methylation of Cs in all contexts (CpG, CHG, and CHH). We used the coverage files for all subsequent analyses, and the files were modified by adding two extra columns containing strand and context information, respectively. To obtain reliable methylation estimation, we filtered out the C sites with a sequencing depth lower than 5. Meanwhile, C sites with a sequencing depth higher than 30 were also filtered out based on the sequencing depth distribution. This retained on average 299 million C sites out of 615 million per experiment. We used a binomial test to decide whether a C site was methylated or not. Specifically, using the error rate estimated by the λDNA control, we calculated a *p*-value for each C site according to binomial distribution. The *p*-values were converted to false discovery rates (FDRs) using the Benjamini–Hochberg procedure. We defined an FDR threshold of 0.01. C sites with FDR values lower than 0.01 are regarded as methylated [40]. To measure the overall methylation level of a gene (exons + introns), we used a weighted methylation level [40].

### 2.6. Differential Gene Expression and Methylation of Lab Acclimated Spiders

The raw sequences were quality-checked using FastQC and trimmed using trimmomatic [41], removing the front 10 bases and removing low quality bases using a sliding window. Subsequently, the sequences were run through the so-called new tuxedo protocol [42]. Mapping was achieved with Hisat2 [43], and assembly and merging of the assembled reads was done using Stringtie and Stringtie-merge [44]. For all these steps, the genome annotation for *S. dumicola* was used as reference. Afterwards, Stringtie was used to count the transcripts, thereby obtaining expression values for all transcripts. A table of transcripts for the 199 spiders was retrieved using the R package Ballgown [45]. The expression level per gene per spider individual was measured as fragments per kilobase million (FPKM). For each combination (20 in total) of population and acclimation temperature, we merged the values of the 10 replicates by taking the mean value.

To examine whether the methylation difference in genes between populations could cause expression difference, we tested the correlation (Spearman’s correlation) between the differential expression per gene and the differential methylation per gene. Both measures were calculated between the two populations where the bisulfite sequencing data are available, i.e., Betta and Karasburg (individuals were acclimated under 25 °C, see description above). The differential expression per gene was represented as log2 fold change in FPKM between the two populations. The differential methylation per gene was calculated by subtracting the weighed methylation level between the two populations. Genes with no more than 10 CpG sites sequenced were removed from the analysis. Moreover, because most genes have a very similar methylation level between two populations, we only kept those with the top 5% differential methylation.

### 2.7. DNA Methylation and Stability of Gene Expression

We also tested whether methylated genes tend to have a more stabilized expression. For that, we calculated the standard deviation of the log2 (FPKM) for each gene across the 10 individuals from the Betta population acclimated at 25 degrees. The standard deviation was then compared with the DNA methylation level of each gene of the same 10 individuals. If the stabilizing effect does exist, we could expect higher standard deviation for the lowly methylated genes than for the highly methylated ones.

### 2.8. DNA Methylation in Two Social Stegodyphus Species

CpG _O/E_ was calculated from nucleotide sequence sets of protein coding genes from *S. dumicola* and *S. mimosarum* as (L*#CpG)/(#C*#G), where L is the sequence length, #CpG is the number of CpGs in the region, and #C and #G are the number of Cs and Gs in the region. The distributions of CpG _O/E_ are represented as histograms. Kernel density estimation (KDE) was achieved using the density function in R [46], with a Gaussian-type kernel. KDE was achieved on CpG _O/E_ estimates with zeroes removed, since the low estimate was due to very short genes (data not shown). Normal distributions were fitted to the CpG _O/E_ densities using the R function normalmixEM [47]. In order to identify putative ortholog protein coding genes between the two species, we used the reciprocal best blast hits approach. tblastx was performed among protein coding nucleotide sequences of *S. dumicola* (this study) and *S. mimosarum* [48], and we obtained 10,233 putative ortholog genes. As a proxy for the historical DNA methylation level, we estimated CpG _O/E_ for the set of ortholog genes. We used PRANK [49] to align the set of ortholog sequences (translated alignment version -translate). We only kept codons that we included in 60 bp stretches that had at most 10 positions that were not identical (SNP and gaps were counted as not identical). Only alignments longer than 180 bp were kept (9128 in total). To test if the exon level DNA methylation is evolutionarily conserved between *S. mimosarum* and *S. dumicola*, we calculated Pearson’s correlation coefficient by correlating CpG _O/E_ estimates of the ortholog genes of two species. We estimated the *dN/dS* ratio (the ratio of the number of nonsynonymous substitutions per nonsynonymous site to the number of synonymous substitutions per synonymous site) as a measure of evolutionary rate for each gene using PAML version 4.6 [50]. Pearson’s correlation coefficient was calculated by correlating *dN*, *dS*, *dN*/*dS*, and average CpG _O/E_ estimates of the two species. 

### 2.9. Comparative Analyses of DNA Methylation in Chelicerates

We downloaded genome sequences and protein coding nucleotide sequences from all available chelicerate species for analyses of DNA methylation patterns. We constructed a schematic cladogram of the chelicerate species included in this study. The phylogenetic relationships of the major groups (spiders, scorpions, parasitiformes, acariformes, and horseshoe crabs) are based on the phylogenies published in [48,51]. Grouping of spiders were based on [52], while grouping of parasitiformes and acariformes were based on www.tolweb.org. We also downloaded 15 protein sequences encoded by DNMTs genes in different insect species from Genbank—five DNMT1, five DNMT2, and five DNMT3 (Table S4). We performed blastp analyses to identify putative DNMTs in chelicerate species whose genome has been sequenced and protein coding genes annotated (Table S5). The threshold Expected (e-) value was set to e-10. In addition, we blasted (tblastn) the insect DNMTs to the chelicerate genomes without a gene annotation, to identify potentially functional DNMTs. In the same way, we looked for DNMT1 in the tick *Rhipicephalus microplus*. Subsequently, the identified sequences were blasted (blastp) using Web BLAST against the non-redundant protein database (nr) database at NCBI to verify that they were members of the DNMT family, and to hypothesize if they belong to DNA methyltransferase subfamily DNMT1, DNMT2 or DNMT3. All DNMTs have a conserved catalytic DNA methylase domain, while the different types of DNMTs have additional characteristic conserved domains [53]. We predicted conserved protein domain structures using SMART (Normal mode) [54], to further support that the sequences are DNMTs and belong to the DNMT1, DNMT2 or DNMT3 subfamilies. The unique CFT motif of DNMT2s were manually annotated [55]. We used the ClustalW algorithm [56] to align DNMTs followed by manual adjustments. It was not possible to align the different DNMT types, and three separate alignments were produced. Separate phylogenies of the three DNMT types were produced using the neighbor-joining algorithm (JJT) in Mega 7.0 [57].

We examined the occurrence of DNA methylation in the genomes of sequenced chelicerate species using the measure CpG _O/E_ as a proxy. We calculated CpG _O/E_ for protein-coding gene sequences and the entire genome, separately. CpG _O/E_ for genes was calculated as in the former analysis, while CpG _O/E_ for the genome was calculated by splitting the genome into 1000 bp fragments and calculating CpG _O/E_ per fragment. As a control, we also calculated GpC _O/E_ ((L*#GpC)/(#C*#G)).

## 3. Results

### 3.1. Genome Assembly and Annotation

K-mer depth distribution analysis using SOAPdenovo2-r240 [58] suggests that the actual genome size is around 4.29 Gb (Figure S4). The genome of *S. dumicola* was de novo assembled by a combination of 70× coverage short-read paired-end Illumina sequencing (an insert size of 300 bp) and 9× coverage long-read PacBio sequencing. A total of 2.55 Gb were assembled into 16,532 scaffolds with an N50 of 254,130 bp (Table 1). The GC content was estimated to be 33.3%. The BUSCO analysis showed that the functional completeness of the genome is quite good. Of the 1066 orthologs recorded in the Arthropoda_odb9 database, 976 (91.6%) were found to be present in our assembly. As an additional test of assembly quality, we mapped back Illumina data to the produced assembly. When the “bwa mem” function was used, 95.83% of the reads were mapped, and 86.33% were properly paired. When “bwa aln -n 2” was used, 78.87% of the reads were mapped, and 73.36% were properly paired. The difference between the two rounds of mapping suggests a high portion of repetitive sequences in the genome. The high mapping rate of the first round indicates the non-repetitive regions are well assembled. The depth distribution and the insert size distribution are unimodal (Figure S5) and are nearly identical between the two rounds. The depth distribution plot peaks at 60, suggesting an actual genome size of 4.37 Gb, corroborating the estimation from the K-mer distribution plot.

Retrained AUGUSTUS predicted 37,601 gene models in our assembly. Of these gene models, 16,450 had support from RNA data, while 6649 were found in repetitive regions. A total of 1769 transcripts that were not predicted by AUGUSTUS were assembled from RNA sequence data by Stringtie, and were added to the final list of gene models (protein and nucleotide sequences and an annotation (gff) file can be downloaded as Supplementary Data). Furthermore, 92.38% of the exons (n = 141,176) discovered with the transcriptome data were predicted by AUGUSTUS (Figure S6), indicating a high quality of prediction. About 51% of the genome assembly consists of a repetitive sequence. About half of the repetitive DNA are TEs (LINEs, SINEs, LTRs, and DNA elements), while the other half is unclassified (Table S6). The extent and composition of repetitive sequences in *S. dumicola* is similar to what was reported in the closely related *S. mimosarum* [48]. See Table 1 for a summary of the assembly and annotation.

### 3.2. Methylation Pattern in Stegodyphus dumicola

**Figure 1:**
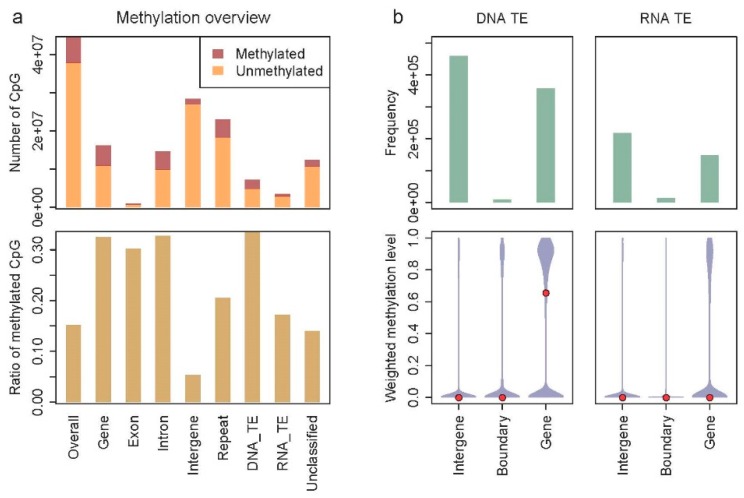
Distribution of CpG DNA methylation across the genome of *Stegodyphus dumicola*. (**a**) The upper part shows the numbers of CpGs and the lower part shows the relative DNA methylation of CpGs in different genomic elements. The genomic elements are nested: “Overall” covers the entire genome, “Gene” covers exons and introns, while “Repeat” covers DNA TEs (DNA_TE), RNA TEs (RNA_TE), and unclassified repeats. (**b**) DNA methylation of CpGs located in DNA and RNA TEs. The upper part shows the number of TEs located in “Intergenic regions,” in “Intergenic-genic boundaries,” and in “Genes.” The lower part shows the distribution of the methylation level in the three different categories. The medians are shown with red dots.

The WGBS mapping rate was 49% for the Betta population and 48% for the Karasburg population. We found most DNA methylation in *S. dumicola* in CpG context, and only little in CHH and CHG contexts. About 15% of the cytosines in CpG context were methylated. Only 0.017% of the cytosines in CHH context and 0.018% of the cytosines in CHG context were not converted during bisulfite treatment. About a third of the CpGs in genes were methylated, while only 5% of the intergenic CpGs were methylated (Figure 1a). Exons and introns were methylated to more or less the same extent (Figure 1a). DNA TEs were on average methylated to the same extent as gene bodies or even a bit higher when about 35% of CpGs were methylated (Figure 1a). They were hypo-methylated when located in intergenic regions, but highly methylated when located within genes (exons and/or introns) (Figure 1b). A similar pattern was found for RNA TEs, except that their average methylation level was somewhat lower (about 17% of CpGs being methylated) (Figure 1a,b). TEs located within genes showed a similar methylation status as the gene itself; un-methylated genes carried un-methylated TEs and methylated genes carried methylated TEs (Figure S7). Within the Karasburg population, individuals showed extremely similar methylation patterns (data not shown).

#### 3.2.1. DNA Methylation and Gene Expression

**Figure 2:**
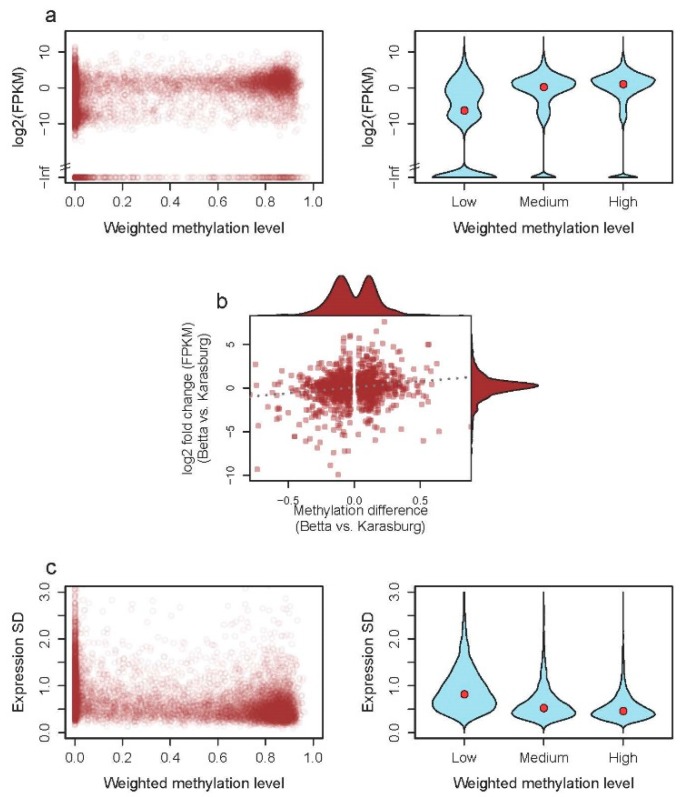
The association between DNA methylation and gene expression in *S. dumicola*. (**a**) The relationship between gene DNA methylation and gene expression in individuals from the Betta population. Left part, the level of DNA methylation and gene expression (FPKM) is plotted (open circles) per gene. Right part, the same data, but categorized into three categories of DNA methylation levels (low [0–0.2]; medium [0.2–0.7]; high [0.7–1]). All three categories are pairwise significantly different (Wilcoxon rank-sum test, *p* < 2 × 10^−16^). (**b**) Differential gene expression as a function of differential DNA methylation. Expression and DNA methylation differences were estimated and averaged across 10 individuals from each of 10 nests from Betta (B25) and Karasburg (K25) that were acclimated at 25 degrees in the lab for 6 weeks. Each dot represents a gene that passed filtering (see Methods Section 2.6). The dotted grey line is shown to highlight the trend in the data. Spearman’s correlation results in *rho* = 0.11 and *p* = 5.6 × 10^−5^. (**c**) The relationship between the standard deviation (SD) of gene expression (log2 (FPKM)) and the DNA methylation coverage ratio of the 10 individuals from the Betta population acclimated at 25 degrees. Only genes that were expressed in all individuals are included.

We found that, within the *S. dumicola* genome, genes that were not methylated on average had a lower expression than genes that were methylated to some extent (all pairwise tests: Wilcoxon rank-sum test, *p* < 2 × 10^−16^) (Figure 2a). When comparing differences in DNA methylation level to differences in expression level among individuals from the Betta and Karasburg populations, but acclimated to same temperature in the lab, we found a weak but significantly positive correlation (Spearman’s correlation, *rho* = 0.11, *p* = 5.6 × 10^−5^) (Figure 2b).

#### 3.2.2. DNA Methylation and Stability of Expression

The expression of genes that were not methylated varies significantly more among individuals compared to genes that were methylated (all pairwise tests: Wilcoxon rank-sum test, *p* < 2 × 10^−16^) (Figure 2c). This pattern is true when only genes that are expressed in all individuals are considered (Figure 2c), but also when all genes that are expressed in at least one individual are considered (Figure S8).

#### 3.2.3. DNA Methylation in Two Social *Stegodyphus* Species

**Figure 3:**
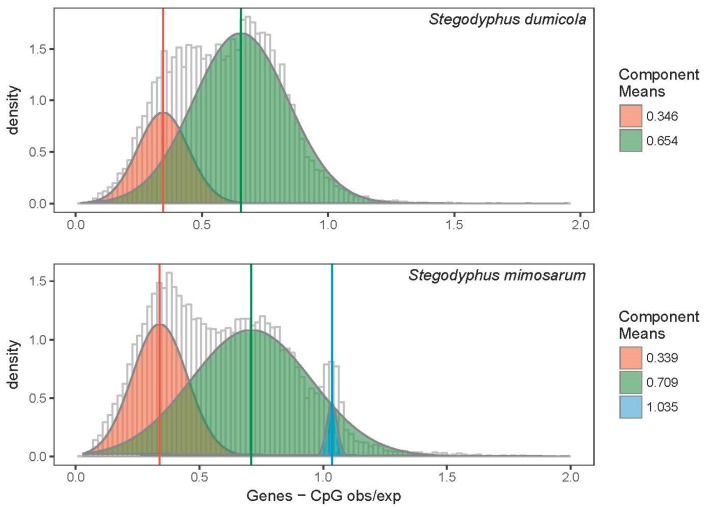
Frequency distribution of CpG _O/E_ in protein coding genes of two social *Stegodyphus* species, *S. dumicola* and *S. mimosarum*. Predicted protein coding genes located in repetitive regions were excluded. Normal distributions were fitted to the data using the EM algorithm for normal mixtures. For *S. dumicola*, Peak 1 had a mean = 0.35 and a standard deviation = 0.10, while Peak 2 had a mean = 0.65 and a standard deviation = 0.19. For *S. mimosarum*, Peak 1 had a mean = 0.34 and a standard deviation = 0.11, Peak 2 had a mean = 0.71 and a standard deviation = 0.24, and Peak 3 had a mean = 1.04 and a standard deviation = 0.02.

Indirect measures of DNA methylation in both social *Stegodyphus* species provide evidence of groups of genes being differently methylated, as indicated by more than one CpG _O/E_ peak (Figure 3). In *S. mimosarum*, three peaks were observed, and this has has not been found in any other species. Only two peaks were observed in *S. dumicola*. The three identified *S. mimosarum* peaks had means of 0.34, 0.71, and 1.04, respectively, while the two identified *S. dumicola* peaks had means of 0.35 and 0.65 (Figure 3). This difference among the two social *Stegodyphus* species may reflect real differences in DNA methylation status or that gene annotation pipelines differ. A total of 9128 ortholog genes that formed alignments longer than 180 bp were identified among *S. dumicola* and *S. mimosarum* by best reciprocal blast analysis. We found a strong and highly significant correlation between CpG _O/E_ estimates in ortholog genes in *S. mimosarum* and *S. dumicola* (Pearson’s rho = 0.78 (0.77–0.79), *p* < 10^−16^) (Figure S9). Evolutionary rates (*dS*, *dN*, and *dN*/*dS*) were additionally found to have significantly positive correlations to CpG _O/E_ (Figure S10).

### 3.3. Comparative Analyses of DNA Methylation in Chelicerates

In chelicerate species with an annotated genome, the number of copies of DNMTs was recorded (Table S7). The spider species had copies of all DNMTs, except for *Loxoceles reclusa* for which no DNMTs were identified. The scorpion (*Centruroides sculpturatus*) and horseshoe crab (*Limulus polyphemus*) also have copies of all DNMTs. In Acariformes and Parasitiformes, the pattern shows that different species have copies of different DNMTs. The DNMT2 protein was the most commonly found DNMT among the studied chelicerates. Domain structures were predicted in all DNMT protein sequences (Figure S11). All predicted domains were consistent with the hypothesized DNMT grouping. Cases where domains were expected, but not predicted, may be explained by incomplete sequences. The three different DNMT types could only be aligned with sequences of the same type, which was achieved for the chelicerate DNMT sequences and a number of insect sequences of each type (Figure S12). The estimated phylogenetic relationships show that the insect DNMTs form monophyletic groups for all three DNMT types, suggesting that the variation among chelicerate DNMTs originate from after the split with insects (Figure S12).

**Figure 4:**
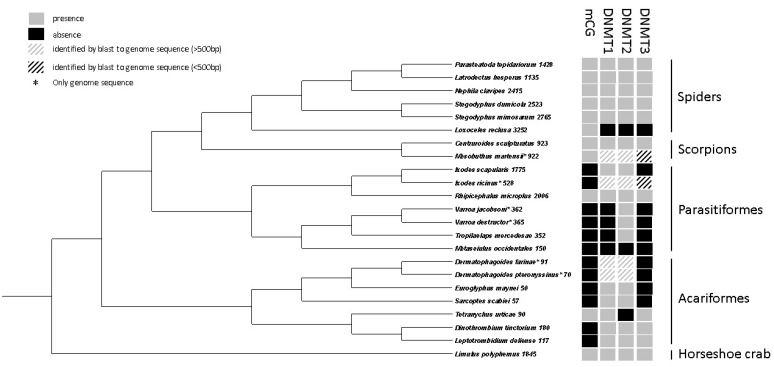
The presence/absence of indirectly inferred DNA methylation (CpG _O/E_), DNMT1, DNMT2, and DNMT3 in previously sequenced chelicerate genomes. The cladogram was schematically put together from several lines of evidence (see Materials and methods Section 2.9.).

Most species that have evidence of CpG methylation (a CpG _O/E_ peak below 1) also carried one or more copies of DNMT3 (Figure 4, Figure S13). *L. reclusa* is an exception, as surprisingly no DNMTs were identified in this species. The two closely related Acariformes, *Dinothrombium tinctorium* and *Leptotrombidium deliense*, are also exceptions, since they carried a DNMT3 copy, but did not show evidence of CpG methylation (Figure 4, Figure S13).

## 4. Discussion

The number of species representing different taxa that have their genome bisulfite sequenced is increasing rapidly, and patterns of DNA methylation and information on its functional role is emerging, both within and across individual genomes, and among taxonomical groups. Previous results demonstrate that DNA methylation patterns among taxa are conserved, which is consistent with an ancient origin and important roles of DNA methylation. However, evidence also suggests that DNA methylation level and its distribution and function may be evolutionarily labile. Nonetheless, knowledge about DNA methylation patterns and function is still patchy across animal taxa, and very little information is available on, for example, chelicerate species. We here present the first whole genome bisulfite sequencing of a chelicerate species: the spider species *S. dumicola*.

In *S. dumicola*, the DNA methylation level is relatively high, mainly found in cytosines in CpG context, and methylations are concentrated in genes (genome average: about 15% of all CpGs; genes: about 33%). This overall pattern is similar to what is found in other invertebrate species in which DNA methylation has been studied, while the amount of DNA methylation are among the highest reported in invertebrates and equals fx Blattodea species [9]. On the other hand, the finding that methylations are concentrated in gene bodies in invertebrates, is in contrast to DNA methylation patterns found in vertebrate species where the genomes are commonly globally methylated [1], and corroborate the observation of a major evolutionary transition of DNA methylation pattern across the invertebrate–vertebrate boundary [1]. The molecular machinery of DNMTs in animals has an ancient evolutionary origin that predates the common ancestor of animals [55,59,60], but the resulting DNA methylation patterns and proposed functions of DNA methylation vary among animal taxa. The aforementioned evolutionary transition at the vertebrate–invertebrate boundary could have caused a functional divergence, especially since methylation of TEs in vertebrates are believed to limit their proliferation [61]. TEs seem hypo-methylated in many invertebrates [62,63], but no evidence supports a functional divergence.

We found a substantial level of methylation in TEs, especially DNA elements. However, TEs located in intergenic regions show much lower methylation levels than those located within genes. Similar results have been reported in the marbled crayfish [8]. One possible explanation is that the higher methylation levels of TEs within genes is a byproduct of the gene methylation process. Alternatively, proliferation of TEs within genes constitute a greater risk [64,65], and DNA methylation serves to silence TEs located within genes. Our finding that methylated TEs within genes are found almost exclusively in genes that are also methylated supports the byproduct explanation. However, the finding that DNA TEs located within genes are methylated even more than the gene itself opens up the possibility of a specific functional role of DNA transposon methylation, at least when located in genes.

The functions of gene DNA methylation in invertebrates is not yet fully understood; however, some studies provide correlative evidence consistent with the regulation of gene expression as a function [6,8,66], while other studies do not find an association [67,68]. The DNA methylation level of genes across individual invertebrate genomes often varies substantially, and our results show that methylated genes are more highly expressed than low- or un-methylated genes. This is also supported by results in other species [6]. It was recently suggested that an additional function of DNA methylation might be to stabilize gene expression [8], so that genes whose expression are important across ecological contexts are methylated to minimize fluctuations of their expression level. Our finding that un-methylated genes in *S. dumicola* vary much more in their expression among populations and acclimation temperatures than methylated genes do (Figure 2C) supports this hypothesis. The result that the DNA methylation level among genes correlates with evolutionary rates is consistent with the hypothesis that housekeeping genes are among the most methylated [6,14] and under stronger selective constraints compared to other genes [15,16]. However, this is not a universal pattern, and for example in the *Nasonia* genus such a correlation was not found [69].

Differential DNA methylation among individuals or populations has recently been hypothesized to influence adaptation via adaptive gene regulation [70,71,72]. If so, an adaptive response caused by DNA methylation may either be plastic and based on environmentally induced DNA methylation, or evolutionary and based on inherited DNA methylation. For example, studies on fish have shown that DNA methylation levels can be highly plastic under different environmental regimes [73,74]. Such effects may lead to the divergence of DNA methylation across populations and potentially to transgenerational adaptive responses if inherited [5]. We document a significant positive correlation between differential expression and differential DNA methylation among populations; however, only a small part of the variation in differential expression can be explained by DNA methylation. There can be many reasons for this small effect of DNA methylation on gene expression, and most likely it results from several and not mutually exclusive explanations: (1) the general effect of methylation on expression is small, (2) other mechanisms regulate gene expression as well, (3) DNA methylation only affects gene expression in a subset of genes, or (4) DNA methylation also plays other roles, such as guiding alternative splicing [75] and stabilizing gene expression, as suggested above [8]. It is important to note that it is not clear whether the observed correlation between differential expression and differential DNA methylation among populations is due to irreversible environmentally induced DNA methylation, or inherited differences among populations.

Adaptive gene regulation is naturally of great importance to most organisms that live in changing or heterogeneous environments, either as plastic or evolutionary responses. Especially organisms that are limited in their behavioral responses to avoid environmental stresses, and organisms with low genetic diversity and therefore low evolutionary potential, may need to rely on gene regulatory adaptations. Social spider species such as *S. dumicola* that live their entire life in family groups at a stationary nest [22] may have only limited opportunities to behaviorally avoid, for example, humidity and temperature stress. In addition, their social behavior and associated traits have resulted in extremely low genetic diversity across their entire species range [25]. For those reasons, adaptive gene regulation based on DNA methylation is potentially especially important in social spiders. A similar situation exists in the marbled crayfish (*Procambarus virginalis*) that is parthenogenetic. Epigenetic diversity has been shown to be larger than genetic diversity in this species [8,76], and the same genotype can express different phenotypes dependent on developmental conditions [77], opening the possibility that epigenetic differences may underlie adaptive phenotypes.

Within arthropods, DNA methylation patterns have primarily been studied in insects, where the extent of DNA methylation has been found to vary substantially [9]. In some insect orders (Hemiptera and Blattodea), DNA methylation levels are found to be relatively high with up to 40% of the CpGs in coding sequences being methylated, while DNA methylation is lost in Diptera species, and intermediate levels of DNA methylation is reported in other orders [9]. We performed a phylogenetic analysis and document high variation in the presence/absence of DNA methylation between different taxonomic groups of chelicerates. In both Parasitiformes and Acariformes, most species seem to have lost DNA methylation, while all spiders, scorpions, and horseshoe crabs included show evidence of DNA methylation. While the loss of DNA methylation in insects is explained well by loss of the DNMT1 gene, the explanation is not as clear in chelicerates. All species studied that show evidence of DNA methylation also have gene copies of both DNMT1 and DNMT3, except for the spider *L. reclusa*, where neither DNMT1 nor DNMT3 were identified. For the species that have lost DNA methylation, some have lost both DNMT1 and DNMT3, some only DNMT1, and some either DNMT1 or DNMT3 (Figure 4).

## 5. Conclusions

The first DNA methylation study in a chelicerate species shows that DNA methylation occurs mainly in CpG context in genes. Our results are consistent with DNA methylation in *S. dumicola*, playing a role in the regulation of both the level and the stability of gene expression. However, as we demonstrate correlative associations, the causal relationships are still to be determined. Furthermore, comparative phylogenetic analysis of DNA methylation patterns shows that most chelicerate species, whose genomes have been sequenced, have DNA methylation, but also that it has been lost several times.

## Figures and Tables

**Table 1:** Summary of genome assembly.

Estimated genome length	4,287,877,091
Sequence coverage	50
Assembled genome length	2,551,871,755
Number of sequences	16,532
N50	254,130
Largest	1,740,957
GC content	3326%
Number of protein-coding genes	37601
Exon length	381
Intron length	5453
Repeat content	51,41%
RNA TEs	11,4%
DNA TEs	1487%
Unclassified	2396%

TE: transposable element.

## Supplementary Materials

The following are available online at https://www.mdpi.com/2073-4425/10/2/137/s1, Figure S1: Map of populations, Figure S2: Genome assembly depth-length correlation, Figure S3: Methylation mapping depth and insert size distributions, Figure S4: K-mer depth distribution, Figure S5: Mapping depth and insert size distributions of illumina data mapped to the genome assembly, Figure S6: RNA-seq exons recovered by Augustus, Figure S7: Weighted methylation level correlation between genes and TEs, Figure S8: Correlations between expression variation and methylation level in genes expressed in at least one individual, Figure S9: CpG _O/E_ correlation between *S. mimosarum* and *S. dumicola*, Figure S10: Correlations between CpG _O/E_ and evolutionary rates, Figure S11: Predicted domain structures in DNMT sequences, Figure S12: Phylogenies based on DNMTs, Figure S13: CpG _O/E_ and GpC _O/E_ density plots, Table S1: GPS coordinates for the sampled populations, Table S2: Optimization parameters for the genome assembly, Table S3: Bisulfite sequencing and mapping results summary, Table S4: Insect DNMT’s used for blast analysis, Table S5: Chelicerate species included in the comparative analysis of DNA methylation patterns, Table S6: RepeatMasker analysis results, Table S7: Number of gene copies of DNMT’s identified in species with annotated genomes. Also included the R script the gene annotation, protein.fasta and transcript.fasta files.

## Data Accessibility

Data can be found at Genbank: Bioproject PRJNA510316.

## References

[1] Keller T.E., Han P., Yi S.V. (2016). Evolutionary transition of promoter and gene body DNA methylation across invertebrate-vertebrate boundary. Mol. Biol. Evol..

[2] Varriale A. (2014). DNA Methylation, Epigenetics, and evolution in vertebrates: Facts and challenges. Int. J. Evol. Biol..

[3] Geiman T.M., Muegge K. (2010). DNA methylation in early development. Mol. Reprod. Dev..

[4] Flores K.B., Wolschin F., Amdam G.V. (2013). The role of methylation of DNA in environmental adaptation. Integr. Comp. Biol..

[5] Heard E., Martienssen R.A. (2014). Transgenerational epigenetic inheritance: Myths and mechanisms. Cell.

[6] Sarda S., Zeng J., Hunt B.G., Yi S.V. (2012). The evolution of invertebrate gene body methylation. Mol. Biol. Evol..

[7] Kvist J., Athanasio C.G., Solari O.S., Brown J.B., Colbourne J.K., Pfrender M.E., Mirbahai L. (2018). Pattern of DNA methylation in *Daphnia*: Evolutionary perspective. Genome Biol. Evol..

[8] Gatzmann F., Falckenhayn C., Gutekunst J., Hanna K., Raddatz G., Carneiro V.C., Lyko F.F. (2018). The methylome of the marbled crayfsh links gene body methylation to stable expression of poorly accessible genes. Epigenet. Chromatin.

[9] Bewick A.J., Vogel K.J., Moore A.J., Schmitz R.J. (2017). Evolution of DNA methylation across Insects. Mol. Biol. Evol..

[10] Moore L.D., Le T., Fan G.P. (2013). DNA methylation and its basic function. Neuropsychopharmacology.

[11] Jeltsch A., Ehrenhofer-Murray A., Jurkowski T.P., Lyko F., Reuterd G., Ankri S., Nellen W., Schaefer M., Helm M. (2017). Mechanism and biological role of Dnmt2 in nucleic acid methylation. RNA Biol..

[12] Law J.A., Jacobsen S.E. (2010). Establishing, maintaining and modifying DNA methylation patterns in plants and animals. Nat. Rev. Genet..

[13] Beeler S.M., Wong G.T., Zheng J.M., Bush E.C., Remnant E.J., Oldroyd B.P., Drewell R.A. (2014). Whole-genome DNA methylation profile of the Jewel Wasp (*Nasonia vitripennis*). G3-Genes Genomes Genet..

[14] Suzuki M.M., Bird A. (2008). DNA methylation landscapes: Provocative insights from epigenomics. Nat. Rev. Genet..

[15] Duret L., Mouchiroud D. (2000). Determinants of substitution rates in mammalian genes: Expression pattern affects selection intensity but not mutation rate. Mol. Biol. Evol..

[16] Pal C., Papp B., Hurst L.D. (2001). Highly expressed genes in yeast evolve slowly. Genetics.

[17] Takuno S., Gaut B.S. (2013). Gene body methylation is conserved between plant orthologs and is of evolutionary consequence. Proc. Natl. Acad. Sci. USA.

[18] Grbic M., Van Leeuwen T., Clark R.M., Rombauts S., Rouze P., Grbic V., Osborne E.J., Dermauw W., Phuong C.T.N., Ortego F. (2011). The genome of *Tetranychus urticae* reveals herbivorous pest adaptations. Nature.

[19] Duncan B.K., Miller J.H. (1980). Mutagenic deamination of cytosine residues in DNA. Nature.

[20] Pfeifer G.P. (2006). Mutagenesis at methylated CpG sequences. DNA Methylation Basic Mech..

[21] Settepani V., Bechsgaard J., Bilde T. (2016). Phylogenetic analysis suggests that sociality is associated with reduced effectiveness of selection. Ecol. Evol..

[22] Lubin Y., Bilde T. (2007). The evolution of sociality in spiders. Adv. Study Behav..

[23] Vanthournout B., Busck M.M., Bechsgaard J., Hendrickx F., Schramm A., Bilde T. (2018). Male spiders control offspring sex ratio through greater production of female-determining sperm. Proc. R. Soc. B-Biol. Sci..

[24] Settepani V., Bechsgaard J., Bilde T. (2014). Low genetic diversity and strong but shallow population differentiation suggests genetic homogenization by metapopulation dynamics in a social spider. J. Evol. Biol..

[25] Settepani V., Schou M.F., Greve M., Grinsted L., Bechsgaard J., Bilde T. (2017). Evolution of sociality in spiders leads to depleted genomic diversity at both population and species levels. Mol. Ecol..

[26] Leffler E.M., Bullaughey K., Matute D.R., Meyer W.K., Segurel L., Venkat A., Andolfatto P., Przeworski M. (2012). Revisiting an old riddle: What determines genetic diversity levels within species?. PLoS Biol..

[27] Ye C.X., Hill C.M., Wu S.G., Ruan J., Ma Z.S. (2016). DBG2OLC: Efficient assembly of large genomes using long erroneous reads of the third generation sequencing technologies. Sci. Rep..

[28] Ye C.X., Ma Z.S.S., Cannon C.H., Pop M., Yu D.W. (2012). Exploiting sparseness in de novo genome assembly. BMC Bioinform..

[29] Walker B.J., Abeel T., Shea T., Priest M., Abouelliel A., Sakthikumar S., Cuomo C.A., Zeng Q.D., Wortman J., Young S.K. (2014). Pilon: An integrated tool for comprehensive microbial variant detection and genome assembly improvement. PLoS ONE.

[30] Simao F.A., Waterhouse R.M., Ioannidis P., Kriventseva E.V., Zdobnov E.M. (2015). BUSCO: Assessing genome assembly and annotation completeness with single-copy orthologs. Bioinformatics.

[31] Li H., Durbin R. (2009). Fast and accurate short read alignment with Burrows-Wheeler transform. Bioinformatics.

[32] Stanke M., Diekhans M., Baertsch R., Haussler D. (2008). Using native and syntenically mapped cDNA alignments to improve de novo gene finding. Bioinformatics.

[33] Smit A.F.A., Hubley R., Green P. RepeatMasker Open-4.0. 2013–2015. http://www.repeatmasker.org.

[34] Benson G. (1999). Tandem repeats finder: A program to analyze DNA sequences. Nucleic Acids Res..

[35] Bao Z.R., Eddy S.R. (2002). Automated de novo identification of repeat sequence families in sequenced genomes. Genome Res..

[36] Price A.L., Jones N.C., Pevzner P.A. (2005). De novo identification of repeat families in large genomes. Bioinformatics.

[37] Krueger F., Andrews S.R. (2011). Bismark: A flexible aligner and methylation caller for Bisulfite-Seq applications. Bioinformatics.

[38] https://github.com/s-andrews/FastQC.

[39] https://github.com/FelixKrueger/TrimGalore.

[40] Schultz M.D., Schmitz R.J., Ecker J.R. (2012). ‘Leveling’ the playing field for analyses of single-base resolution DNA methylomes. Trends Genet..

[41] Bolger A.M., Lohse M., Usadel B. (2014). Trimmomatic: A flexible trimmer for Illumina sequence data. Bioinformatics.

[42] Pertea M., Kim D., Pertea G.M., Leek J.T., Salzberg S.L. (2016). Transcript-level expression analysis of RNA-seq experiments with HISAT, StringTie and Ballgown. Nat. Protoc..

[43] Kim D., Landmead B., Salzberg S.L. (2015). HISAT: A fast spliced aligner with low memory requirements. Nat. Methods.

[44] Pertea M., Pertea G.M., Antonescu C.M., Chang T.C., Mendell J.T., Salzberg S.L. (2015). StringTie enables improved reconstruction of a transcriptome from RNA-seq reads. Nat. Biotechnol..

[45] Fu J., Frazee A.C., Collado-Torres L., Jaffe A.E., Leek J.T. (2018). Ballgown: Flexible, Isoform-Level Differential Expression Analysis.

[46] Team R.C. (2018). R: A Language and Environment for Statistical Computing.

[47] Benaglia T., Chauveau D., Hunter D.R., Young D.S. (2009). mixtools: An R Package for analyzing finite mixture models. J. Stat. Softw..

[48] Sanggaard K.W., Bechsgaard J.S., Fang X.D., Duan J.J., Dyrlund T.F., Gupta V., Jiang X.T., Cheng L., Fan D.D., Feng Y. (2014). Spider genomes provide insight into composition and evolution of venom and silk. Nat. Commun..

[49] Loytynoja A., Goldman N. (2008). Phylogeny-aware gap placement prevents errors in sequence alignment and evolutionary analysis. Science.

[50] Yang Z.H. (2007). PAML 4: Phylogenetic analysis by maximum likelihood. Mol. Biol. Evol..

[51] Schwager E.E., Sharma P.P., Clarke T., Leite D.J., Wierschin T., Pechmann M., Akiyama-Oda Y., Esposito L., Bechsgaard J., Bilde T. (2017). The house spider genome reveals an ancient whole-genome duplication during arachnid evolution. BMC Biol..

[52] Wheeler W.C., Coddington J.A., Crowle L.M., Dimitrov D., Goloboff P.A., Griswold C.E., Hormiga G., Prendini L., Ramirez M.J., Sierwald P. (2017). The spider tree of life: Phylogeny of Araneae based on target-gene analyses from an extensive taxon sampling. Cladistics.

[53] Lyko F. (2018). The DNA methyltransferase family: A versatile toolkit for epigenetic regulation. Nat. Rev. Genet..

[54] Letunic I., Bork P. (2018). 20 years of the SMART protein domain annotation resource. Nucleic Acids Res..

[55] Jurkowski T.P., Jeltsch A. (2011). On the evolutionary origin of eukaryotic DNA methyltransferases and Dnmt2. PLoS ONE.

[56] Thompson J.D., Higgins D.G., Gibson T.J. (1994). Clustal-W—Improving the sensitivity of progressive multiple sequence alignment through sequence weighting, position-specific gap penalties and weight matrix choice. Nucleic Acids Res..

[57] Kumar S., Stecher G., Tamura K. (2016). MEGA7: Molecular Evolutionary genetics analysis version 7.0 for bigger datasets. Mol. Biol. Evol..

[58] Luo R.B., Liu B.H., Xie Y.L., Li Z.Y., Huang W.H., Yuan J.Y., He G.Z., Chen Y.X., Pan Q., Liu Y.J. (2012). SOAPdenovo2: An empirically improved memory-efficient short-read de novo assembler. Gigascience.

[59] Zemach A., Zilberman D. (2010). Evolution of eukaryotic DNA methylation and the pursuit of safer sex. Curr. Biol..

[60] Goll M.G., Bestor T.H. (2005). Eukaryotic cytosine methyltransferases. Annu. Rev. Biochem..

[61] Ikeda Y., Nishimura T., Pontes O., Jin H. (2015). The Role of DNA methylation in transposable element silencing and genomic imprinting. Nuclear Functions in Plant Transcription. Signaling and Development.

[62] Xiang H., Zhu J.D., Chen Q., Dai F.Y., Li X., Li M.W., Zhang H.Y., Zhang G.J., Li D., Dong Y. (2010). Single base-resolution methylome of the silkworm reveals a sparse epigenomic map. Nat. Biotechnol..

[63] Bonasio R., Li Q.Y., Lian J.M., Mutti N.S., Jin L.J., Zhao H.M., Zhang P., Wen P., Xiang H., Ding Y. (2012). Genome-wide and Caste-Specific DNA Methylomes of the Ants *Camponotus floridanus* and *Harpegnathos saltator*. Curr. Biol..

[64] Bewick A.J., Ji L.X., Niederhuth C.E., Willing E.M., Hofmeister B.T., Shi X.L., Wang L., Lu Z.F., Rohr N.A., Hartwig B. (2016). On the origin and evolutionary consequences of gene body DNA methylation. Proc. Natl. Acad. Sci. USA.

[65] Inagaki S., Kakutani T. (2012). What triggers differential DNA methylation of genes and TEs: Contribution of body methylation?. Cold Spring Harb. Symp. Quant. Biol..

[66] Suzuki M.M., Kerr A.R.W., De Sousa D., Bird A. (2007). CpG methylation is targeted to transcription units in an invertebrate genome. Genome Res..

[67] Glastad K.M., Gokhale K., Liebig J., Goodisman M.A.D. (2016). The caste- and sex-specific DNA methylome of the termite *Zootermopsis nevadensis*. Sci. Rep..

[68] Bewick A.J., Sanchez Z., Mckinney E.C., Moore A.J., Moore P.J., Schmitz R.J. (2019). Gene-regulatory independent functions for insect DNA methylation. Epigenetics & Chromatin.

[69] Park J., Peng Z.G., Zeng J., Elango N., Park T., Wheeler D., Werren J.H., Yi S.V. (2011). Comparative analyses of DNA methylation and sequence evolution using Nasonia genomes. Mol. Biol. Evol..

[70] Donohue K. (2014). The epigenetics of adaptation: Focusing on epigenetic stability as an evolving trait. Evolution.

[71] Lind M.I., Spagopoulou F. (2018). Evolutionary consequences of epigenetic inheritance. Heredity.

[72] Danchin E., Charmantier A., Champagne F.A., Mesoudi A., Pujol B., Blanchet S. (2011). Beyond DNA: Integrating inclusive inheritance into an extended theory of evolution. Nat. Rev. Genet..

[73] Metzger D.C.H., Schulte P.M. (2017). Persistent and plastic effects of temperature on DNA methylation across the genome of threespine stickleback (*Gasterosteus aculeatus*). Proc. R. Soc. B-Biol. Sci..

[74] Metzger D.C.H., Schulte P.M. (2018). The DNA methylation landscape of stickleback reveals patterns of sex chromosome evolution and effects of environmental salinity. Genome Biol. Evol..

[75] Maor G.L., Yearim A., Ast G. (2015). The alternative role of DNA methylation in splicing regulation. Trends Genet..

[76] Gutekunst J., Andriantsoa R., Falckenhayn C., Hanna K., Stein W., Rasamy J., Lyko F. (2018). Clonal genome evolution and rapid invasive spread of the marbled crayfish. Nat. Ecol. Evol..

[77] Vogt G., Huber M., Thiemann M., van den Boogaart G., Schmitz O.J., Schubart C.D. (2008). Production of different phenotypes from the same genotype in the same environment by developmental variation. J. Exp. Biol..

